# Tactile Processing and Quality of Sleep in Autism Spectrum Disorders

**DOI:** 10.3390/brainsci11030362

**Published:** 2021-03-12

**Authors:** Dominika Jamioł-Milc, Mirosława Bloch, Magdalena Liput, Laura Stachowska, Karolina Skonieczna-Żydecka

**Affiliations:** 1 Department of Human Nutrition and Metabolomics, Pomeranian Medical University in Szczecin, 71-460 Szczecin, Poland; 2 Sense Therapy Center, Miodowa 57, 71-497 Szczecin, Poland; terapia@integracja-sensoryczna.org; 3 Department of Pharmacognosy and Natural Medicines, Pomeranian Medical University in Szczecin, 70-111 Szczecin, Poland; mliput@pum.edu.pl; 4 Students Scientific Club at the Department of Human Nutrition and Metabolomics, Pomeranian Medical University in Szczecin, 71-460 Szczecin, Poland; l.stachowskaa@gmail.com; 5 Department of Biochemical Sciences, Pomeranian Medical University in Szczecin, 71-460 Szczecin, Poland; karzyd@pum.edu.pl

**Keywords:** disability, autism spectrum disorders, child development, touch, sleep

## Abstract

Individuals with autism spectrum disorders (ASDs) commonly experience problems with the processing of tactile stimuli and poor quality of sleep. The aim of the present study was to analyze whether tactile stimuli modulation (TSM) disorders might be linked to insomnia prevalence in ASD individuals. We hypothesized that sleep disorders in children with ASD may result from improper tactile under/over responsivity. The study included 27 children diagnosed with ASD, aged 6.8 (±2.9 years) with male dominance (*n* = 22, 81.5%). To evaluate the pattern of TSM we used a clinical interview with a parent, and guided and spontaneous observation of the patients. Sleep disorders were diagnosed using the Athens Insomnia Scale. Of all the children diagnosed with TSM, 20 patients (74.1%) had an over-responsivity pattern and 7 children (25.9%) had an under-responsivity pattern. Of the patients, 11 children (40.7%) met the diagnostic criteria for insomnia. The data indicated a statistical tendency for higher prevalence of insomnia in individuals diagnosed with tactile under-responsivity (*p* = 0.051). We concluded that under-responsivity toward tactile stimuli may be partly responsible for poor sleep quality in ASD. There is an urgent need to treat sleep and sensory disruptions which may intensify behavioral difficulties in ASD.

## 1. Introduction

Autism spectrum disorders (ASDs) manifest at an early stage of development, predominantly in males and are comprised of deficits in social interaction, restricted communication skills, and repetitive movements or actions [1,2,3]. The diverse nature of individuals with ASD makes treatment difficult [2]. Furthermore, 85% of individuals with ASD may also be diagnosed with a comorbid disorder, which adds to the complex clinical picture [4]. Although it was found that the majority of individuals with ASD suffer from psychiatric symptoms [2,5], other common comorbidities include gastrointestinal symptoms found in up to 91% of patients with ASD and sleep problems affecting up to 80% of the population [2,6,7].

Sleep is extremely important for humans, even though its role is sometimes neglected. It is a basic biological need of every human being. It predominantly affects health, wellbeing, and daily functioning. Any disruption of sleep architecture impairs physiological pathways within the autonomic nervous system and thereby causes an imbalance in the psychophysical condition of humans, which affects quality of life [8,9]. Sleep is critical for proper neurodevelopment in humans. Circadian rhythm sleep–wake patterns are individual [8,9]. Sleeping habits depend on genetic and environmental factors [10], and differ during particular periods of life, being most important in childhood when intensive brain growth and development take place [11]. Sleep disorders in early life affect health, behavior, and identity in society. It was reported that, in children, ineffective sleep causes irritability, mood swings, and aggression that interfere with daily functioning [12]. Conversely, the risk for developing sleep difficulties was found to be unrelated to the severity of cognitive disability [13]. In children with ASDs, the most common disturbances include insomnia manifesting with parasomnia, sleep breathing disorders, bedtime resistance, difficulty in falling asleep/waking up, and daytime sleepiness [14]. Elrod and Hood conducted actigraphy and polysomnography studies, and their results suggested that individuals with ASDs had a shorter total sleep time, longer sleep latency, and decreased sleep efficiency [15].

Psychological studies state that there is a correlation between sensory processing disorders, behavioral disorders, and sleep–wake disturbances. Results from a study by Sadeh suggest that in individuals with ASD and typically developing children (TDC) with sleep problems, challenging behaviors are more prevalent than in children with no sleep disturbances [16]. Some studies suggest that poor-quality sleep is correlated with more severe ASD symptoms such as exacerbated communication, social skills impairments, and non-functional repetitive behaviors [17]. 

On the other hand, physical, cognitive, and psychosocial domains of every child’s development are influenced by sensory processing patterns [18]. Sensory processing is a concept characterizing the processes of reception, organization, and modulation of stimuli flowing from the outside world in order to produce automatic and adaptive responses to specific sensory information. The efficiency of sensory processing is therefore crucial for the broad welfare of a child [19]. Improper sensory experience in ASDs was first noticed by Kanner [20]. Growing evidence about sensory dysfunction in individuals with ASDs [21,22,23] resulted in the inclusion of sensory impairment in the diagnostic criteria of ASDs [24]. Furthermore, it was indicated that core symptoms of ASD are negatively correlated with deficits in sensory processing [25].

The tactile system is the main source of impressions influencing the central nervous system. All tactile pathways intersect in the thalamus, such that information from the left half of the body reaches the right hemisphere of the brain and vice versa. The tactile system performs both defensive and differentiating (discriminative) functions. The functioning of the defense system can be observed immediately after birth, while the differentiating system develops over time. Tactile stimuli also affect human emotional development, the structure of the body schema and self-image, the shaping of free and reflex movements, the induction of postural reflexes, and the creation of a homogeneous perception of the environment. Overall, proper tactile processing provides emotional stability and therefore a good sense of physical and mental health. Deficits within tactile input detection, registration, and interpretation are characteristic of repetitive behaviors and have been identified as overwhelming, confusing, and intensifying [26,27]. Children deprived of tactile stimuli were found to be nervous and restless [28]. Therapeutic touch (massage) in infants makes them more alert and active during the day, improves quality of sleep with fewer awakenings during the night, and hastens the time to fall asleep [29]. 

In light of these facts, the aim of the present study was to analyze whether tactile stimuli modulation (TSM) disorders might be linked to insomnia prevalence in ASD individuals. We hypothesized that sleep disorders in children with ASDs may result from improper tactile under/over responsivity.

## 2. Materials and Methods

### 2.1. Patients

For the purposes of this study, we enrolled 27 children with ASDs, aged 6.8 (±2.9) years, with males predominating (*n* = 22, 81.5%). A diagnosis of ASD was confirmed by a child psychiatrist using ICD-10 (International Statistical Classification of Diseases and Related Health Problems) criteria. For ASDs, the whole ICD-10 F84 category except Rett syndrome was included. All study participants were treated with sensory integration (SI) therapy following the diagnosis made as part of the present study. Parents of the children were familiarized with the purpose of the survey and its course, and all of them gave written informed consent for their children to participate in the project. The study was approved by the Ethics Committee of Pomeranian Medical University, Szczecin, Poland (approval no.: KB-0012/81/15).

### 2.2. Sensory Processing Pattern

The diagnosis of a TSM disorder was made in two 60 min diagnostic meetings. First, two researchers (M.B. and K.S.-Ż.) conducted an interview with a parent. Then the researchers engaged in direct and spontaneous observation of the patient. 

The SI specialist evaluated the subject’s behavior during tactile stimulation of the body (including hands and feet) with different textures. The reactions while playing with plasticine, paints, loose products (rice, flour, grains) were evaluated. Additionally, we checked reaction to touch of the other person (therapist), even from the back, and evaluated any manifestation of touch defense. Hand manipulation during directed play was also examined. We focused on auto-aggressive behavior. Spontaneous observation was performed to discriminate the avoided stimuli while having fun. Overall, the therapist observed the way in which the child responded to stimuli and the child’s ability to generate appropriate adaptive responses to these. In guided observation, the therapist suggested various types of games to the child, which were a source of sensory stimuli. The therapist observed the way of producing appropriate adaptive responses in response to stimuli, while observing the emotional and somatic reactions presented by the child.

The identification of a TSM disorder, namely tactile over-responsivity, tactile under-responsivity, or tactile seeking, was performed according to Miller, Anzalone, Lane, Cermak, and Osten [30]. Due to the intellectual disability of the majority of study participants (*n* = 23), the diagnosis was of a subjective type. A subject was found to be over-responsive (commonly referred to as sensory defensive) if they responded to exact sensation faster and more intensely. Behaviors were impulsive and aggressive. Emotional responses included irritability, moodiness, inconsolability, and poor socialization. Children with tactile under-responsivity disregarded or did not respond to touch stimuli, as if they were not able to detect incoming sensation. Their behaviors indicated apathy, lethargy, and a lack of inner drive to initiate socialization and exploration of the stimuli. Sensory seekers seemed to have a permanent desire for tactile sensation. They easily engaged in actions to achieve more intense sensations. Their behavior included carelessness and restlessness and emotions were overexpressed.

Southern California Sensory Integration Tests, being the gold standard to identify the nature of the processes of SI disorders, were not used in all children due to their severe motor and mental deficits. Only 5 patients were subjected to Finger Identification, Graphestesia, and Localization of Tactile Stimuli tests and scored in accordance with Ayres [31]. These results are available upon request.

At last, the parents completed a questionnaire which asked about their child’s functioning including information regarding pregnancy, childbirth, perinatal circumstances, school history, and sensitivity towards tactile stimuli at home. It collected information from the parent about the child’s preferred activities, their behavior during everyday activities, and the difficulties that the child faced on a daily basis. Spontaneous observation consisted of observing the child’s natural reactions to contact with various sensory stimuli.

### 2.3. Sleep Difficulties

Sleep–wake problems were evaluated using a Polish, validated Athens Insomnia Scale (AIS) consisting of 8 questions [32]. We examined the quality of sleep, in particular: difficulty falling asleep, night and early morning awakenings, total sleep time, and well-being during the next day. The survey was parent-administered. The scale ranged from 0 to 3 (0 = no problems and 3 = very bothersome). The parents were asked to mark each sleep difficulty their children experienced at least three times a week during the last month. In original validation studies it was demonstrated that AIS represents high reliability and validity, and a total score of 6 or more points indicates high probability of insomnia (sensitivity, 93%; specificity, 85%); therefore, a 6 point cut-off was used in the present study [33].

### 2.4. Statistical Methods

Statistical significance was considered at a *p*-value < 0.05. Statistical analyses for this study were performed using the StatView computer software version 5.0 (SAS Institute Inc. Cary, NC, USA). A non-parametric Mann–Whitney test [34] or a Fisher’s exact test [35] was used as appropriate. Using a generalized linear mixed effects model (with a logit link) with age and gender as covariables, we modelled the probability of insomnia by TSM disorder (https://cran.r-project.org/web/packages/ggeffects/vignettes/practical_logisticmixedmodel.html, accessed on 3 January 2021). Using logistic regression, we modelled the probabilities of TSM disorder by AIS score (enter method- all independent variables are entered into the equation at the same time) and clinical data. To control type I errors, the false discovery rate (FDR) approach was used. The calculations were performed using the p.adjust function of the stats package in R (R Foundation for Statistical Computing, Vienna, Austria, https://cran.r-project.org, accessed on 3 January 2021). Power analysis was calculated with G*Power software [36,37].

## 3. Results

### 3.1. Patient Characteristics

Basic information concerning the pregnancy, perinatal, and neonatal periods was collected during the interview with a parent. The median gestational age at birth was 39 hbd (*latin hebdomas*) (Min: 26 hbd; Max: 41.5 hbd). Overall, 11 (40.7%) mothers of autistic individuals declared using pharmacotherapy during pregnancy (predominantly dydrogesterone). A total of 14 (51.8%) children were born vaginally (oxytocin induction in 4). Caesarean section was performed in 13 (48.1%) births. The median weight of newborns was 3400 g (Min: 940 g; Max: 4480 g). Basic characteristics are shown in Table 1.

### 3.2. Sensory Processing Patterns and Sleep Difficulties

By means of interviews and directed and spontaneous observation of the children, TSM was screened in all children included in the study. We identified over-responsivity or under-responsivity toward tactile stimuli in 20 (74.1%) and 7 (25.9%) children, respectively. There were no differences in gestational age (U = 69.0; *p* = 0.95) or birth weight (U = 69.5; *p* = 0.97) between children regarding TSM disorder type. We found no association (*p* > 0.05) between TSM disorder and any other factors as shown in Table 2. We also found no differences in TSM disorders regarding neonatal period complications (*p* < 0.05; Table 2). This was replicated in logistic regression analyses, in which patient age was an independent variable. The full regression output is provided in Supplementary Table S1.

A 6-point cut off in AIS was considered to be associated with insomnia. Using this criteria, there were 11 (40.7%) children with insomnia in the study group (Mean = 5.074 points, SD = 3.61, Median = 5.0, Max = 11). AIS score was not correlated with patients’ age (r = −0.231, *p* = 0.2455).

**Figure 1:**
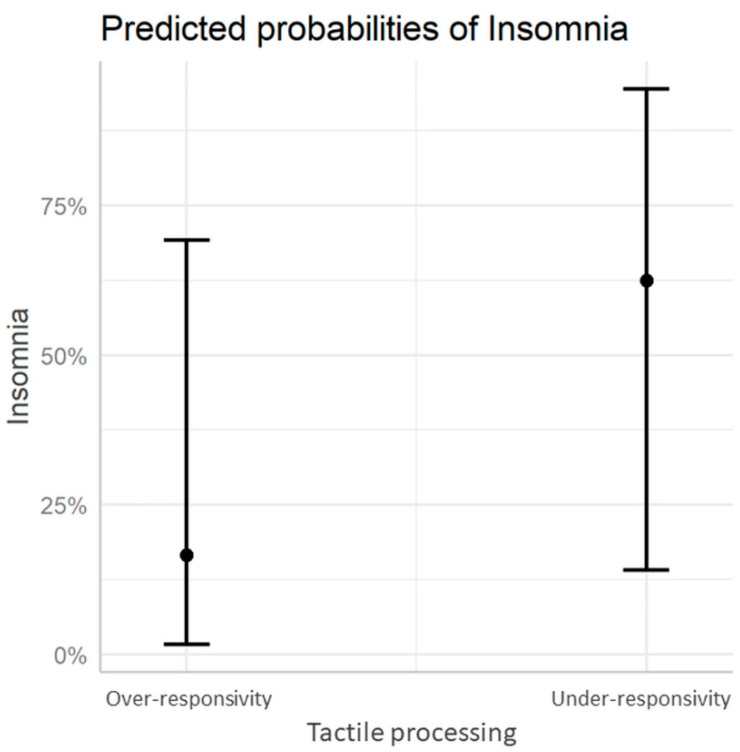
Insomnia Probabilities by TSM disorder, regarding age and gender of patients (*p* = 0.23).

Analysis showed a higher prevalence of insomnia in children with ASD with tactile under-responsivity (*p* = 0.08) (Table 3). The post hoc power of this analysis was 0.56. A priori power analysis depicted that a total sample size of 48 persons with tactile under-responsivity would be needed to achieve acceptable power, specifically 0.8. After taking into account the age and gender of patients, tactile processing was found to potentially increase the prevalence of insomnia (coef. = 2.12, SE = 1.09, *p* = 0.051). The results are shown in Figure 1.

**Figure 2:**
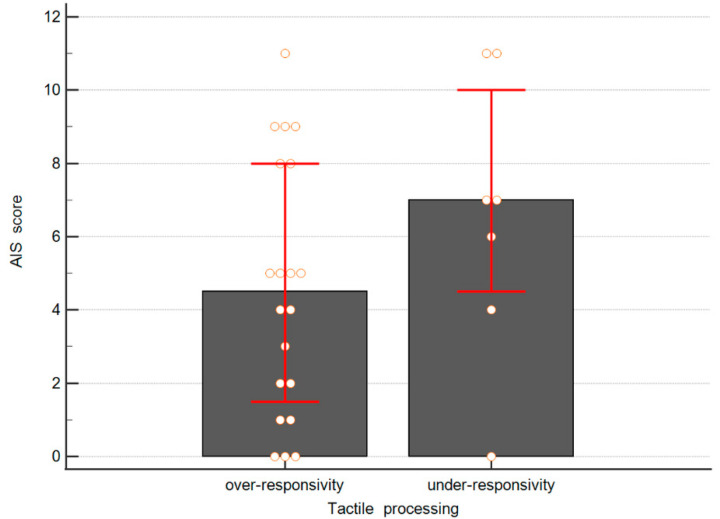
Athens Insomnia Scale (AIS) score by means of tactile processing disorders. Boxes represent medians and bars represent interquartile ranges (*p* = 0.51).

However, when an analysis of TSM disorder prevalence with AIS score was conducted, no association was found (*p* > 0.5), as presented in Figure 2. The post hoc power of this analysis was 0.42. A priori power analysis depicted that a total sample size of 78 persons would be required to achieve suitably powered results.

In the logistic regression model (enter method), we found that the AIS score did not predict tactile under-responsivity (b = 0.16, SE = 0.13, Wald = 1.59, *p* = 0.21, and OR (odds ratio) = 1.18, with 95% CI = 0.9117 to 1.5317).

## 4. Discussion

The present study examined whether TSM disorders might be linked to insomnia prevalence in ASD individuals. We hypothesized that sleep disorders in children with ASD may result from improper tactile under/over-responsivity. The obtained results, although statistically insignificant, indicate that the prevalence of insomnia might be potentially higher in individuals diagnosed with tactile under-responsivity (*p* = 0.051).

Autism spectrum disorders are of varying severity and most often accompanied by other disorders, including psychiatric and somatic ones [38,39]. Sleep disorders are one of the most common psychiatric comorbidities in neurodevelopmental disorders, including ASDs, ADHD, and Down syndrome [40]. In autistic individuals, sleep disorders are common and diverse in nature [41]. In the present study, using AIS, we identified insomnia in over 40% of children included in the study group. This agrees with other studies conducted in the ASDs population. Sleep disruptions have been widely explored in studies based on parental reports. Sivertsen, Posserud, Gillberg, Lundervold, and Hysingin, in a longitudinal total Norwegian population study, concluded that insomnia is ten times more prevalent in ASD patients aged up to 13 years old (y.o.) in comparison to TDC [42]. Souders et al., in a study of 59 American children aged 4–10 y.o. with ASDs, involving parental reports and actigraphy, proved that almost 70% of these children experienced sleep problems [43]. Krakowiak et al. reported that approximately 50% of children with ASDs aged 2–5 y.o. suffered from a sleep problem [44]. Liu, Hubbard, Fabes and Adam enrolled 167 autistic individuals from Phoenix and Tucson (USA) at a mean age of 8.8 y.o., and in 53% of these children identified at least one the following sleep disturbances: bedtime resistance problems, insomnia, parasomnia, sleep disordered breathing, morning arising problems, and daytime sleepiness [45]. Taira, Takase, and Sasaki analyzed the sleep patterns of 89 children with autism from Tokyo [46]. Almost 70% of study participants had various components of insomnia, including difficulty falling asleep and both frequent and early morning awakening. Overall, the literature states that the prevalence of sleep difficulties in ASD children may be as high as 83% [47].

It was demonstrated that sleep disorders trigger greater severity of core behavioral ASD symptoms [48]. It was shown that children with ASDs suffering from sleep problems had greater difficulty in relationships with peers, group membership, and daily functioning [49,50]. Other studies stated that sleep disturbances caused difficulty with concentration and learning problems, and resulted in increased excitability and reactivity [17], as well as restrictive and repetitive behaviors [51].

Studies state that sensory modulation disorders in individuals with ASDs may be extreme [52] wherein sensory under-responsivity was found to be more prevalent in ASDs [53], as sympathetic nervous system arousal was found to be below the baseline level in electrodermal reactivity tests [53]. Furthermore, tactile processing may be one of the most impaired functions in ASDs [54].Touch is essential and phylogenetically the oldest sense of humans. It develops in prenatal life and acts as a basis for the formation and development of other sensory systems. Tactile experience is essential for shaping proper adaptive responses and thereby the physical and mental well-being of the child, expressed by appropriate behavior, including their emotional sphere [55]. Disorders of TSM disrupt the daily functioning of a child, as they produce excessive emotional sensitivity, irritability, or inconsistent emotional reactions [56].

In the present study, all ASD children (*n* = 27, 100%) manifested a tactile processing disorder. The majority of study participants (*n* = 20; 71.4%) exhibited over-response toward tactile stimuli. There were no sensory seekers identified. These findings replicate those of other studies [57,58,59]; however, exact comparisons are difficult due to different demographic and methodological issues. Analysis of the literature showed both over-responsivity and under-responsivity toward tactile stimulation in children with ASDs. Puts, Wodka, Tommerdahl, Mostofsky, and Edden examined 67 individuals with TDC and 32 children of American descent with ASDs, both with a mean age of 10 y.o., in vibrotactile tasks [57]. The authors found that in comparison to TDC, autistic individuals showed elevated static detection thresholds and decreased adaptation and amplitude of discrimination toward tactile stimulation [57]. Additionally, studies in adult individuals with ASDs followed these findings [58,59]. On the other hand, a few studies failed to establish deficits in tactile processing in ASDs [60,61]. In conclusion, the literature analyzing the prevalence of TSM disorders in ASDs is still scarce [62].

In the final step of the present study, we analyzed the relationship between insomnia and TSM pattern in a group of children with ASDs. We observed a higher prevalence of under-response toward tactile stimulation in children with insomnia, however the association was not statistically significant (*p* = 0.051). In turn, Wang et al. indicated a positive correlation (*p* < 0.05) between tactile processing problems and sleep disturbances in preschool children with ASDs (5.18 ± 0.92 y.o., *n* = 81) [63]. In the literature, a number of variables were described as risk factors for disturbed sleep in individuals with ASDs, among them sensory processing disorder [64]. However, there is still too little evidence to state without doubt that these correlates are comorbidities or symptoms of ASDs.

The literature states that the association between sensory processing deficits and sleep alterations exists both in TDC and ASD populations [65], however to the best of our knowledge, the present study is the first to link tactile processing and sleep disruptions in children with ASD originating from Poland. Overall, researchers evaluated the sensory profile globally, not in particular sensory domains, which makes comparisons with this study complicated. Vasak, Williamson, Garden, and Zwicker conducted retrospective analyses in 177 healthy infants and toddlers from Canada, using Sensory Profile and Brief Infant Sleep Questionnaires [66]. The authors found that correlations between sensory seeking and shorter daytime sleep duration, and between hypersensitivity and bedtime resistance, were statistically significant. Roth analyzed the relationship between sleep problems and sensory processing in 22 healthy infants of American descent aged up to 12 months [67]. They discovered that infants under-sensitive to sensory stimuli may have fewer sleep problems. In research by Shochat, Tzischinsky, and Engel-Yeger, tactile sensitivity was a negative prognostic marker for sleep difficulties and, moreover, tactile sensitivity and sensation seeking were more prevalent in TDC with behavioral problems [68].

Similar studies conducted in ASD populations find that sensory hyper-response is predominantly associated with sleep alterations. Research by Mazurek and Petroski aimed to determine whether sensory over-responsivity is associated with poor-quality sleep in children with ASDs [69]. The authors examined 1347 American children and adolescents with ASDs and found that sensory over-responsivity was negatively correlated with sleep quality, although the effect size was small. Reynolds et al. examined the relationship between sensory processing and sleep behaviors in 27 children with ASDs aged between 6 and 12 y.o. by means of electrodermal activity, salivary cortisol, sensory profile questionnaires, and a Child Behavior Checklist [50]. The authors found significant correlation between sensory avoiding behaviors and sleep problems in children with ASDs.

Several studies suggest that all types of sensory modulation disorders cause deficits in social manner, inadequate self-regulation, and skewed competence due to high arousal comprising distraction, impulsiveness, altered activity levels, disorganization, anxiety, and emotional instability [70]. These alterations refer to disruptions within habituation and sensitization mechanisms in the central nervous system [71] and occur as a result of an altered neuronal threshold [72]. Liss et al. postulated that sensory hypo-response, expressed as challenging behavior in ASDs, may be a compensatory mechanism used to lower arousal [70]. This study shows that sleep alterations in ASDs may be explained by tactile under-responsivity. Tzischinsky et al., using the Children’s Sleep Habits Questionnaire (CSHQ) and the complete Sensory Profile, indicated moderate association between sleep alterations and sensory problems in the touch domain among 69 children with autism (4.94 ± 1.23 y.o., 56 male). However, sleep disturbances were more strongly associated with hypersensitivity (over-responsivity) than hyposensitivity (under-responsivity) [73]. This difference in results could be due to the use of different test methods compared to our study.

However, altered behaviors also co-occur with sleep problems in healthy children [16]. Appleyard et al. found a statistically significant positive association (*p* < 0.001) with sleep problems in typically developing 2.5 y.o. toddlers and difficulties in social-relational skills, while longer settling times were related to higher touch sensitivities (*p* = 0.028) [74]. Therefore, it is of great interest to examine whether sleep difficulties in ASD individuals may result from perceptual sensory problems or whether they might be emotional in origin.

Our study has two major flaws. Firstly, the sample size was small. Children that were included in the study were diagnosed with TSM during the first visit the therapy center and the therapy was implemented afterwards. This is the predominant reason for such a small number of autistic individuals being included—all other children were subjected to SI therapy for different periods of time and thus not entered into the study. The second limitation is the lack of a control group. Typically developing children, age- and sex-matched to patients (to adhere correctly to ASDs epidemiological data and tactile sensory development) were not included into the final study, as we designed the study as a cross-sectional one. Lastly, no autistic traits were evaluated.

## 5. Conclusions

Overall, our study indicates that tactile under-responsivity may be a risk factor for sleep difficulties in ASDs. To the best of our knowledge, the present study is the first one conducted in individuals from Poland with ASDs. Our study underlines the need to conduct more research into the ASD population to shed light on the course and pathophysiology of this disorder.

Touch is fundamental to one’s development. It provides a sense of body awareness, and through cooperation with the proprioceptive system, guarantees emotional safety. In ASDs, sensory malfunctions are common and may be extreme. Additionally, sleep problems affect the majority of individuals with ASDs. Oppositional behaviors are core symptoms of the disorder, but may intensify as a consequence of sleep alterations and sensory disruptions. This study underlines:

1. The potential relationship between the tactile stimuli modulation (TSM) pattern and the quality of sleep in ASD individuals.
2. The need to treat sleep and sensory disruptions in ASDs, which may intensify behavioral difficulties.
3. A direction for further research into non-pharmacological treatment for sleep improvement in ASDs.

## Figures and Tables

**Table 1:** Characteristics of the study group (*n* = 27).

Variable	Caesarian Section (*n* = 13) *n* (%)	Vaginal Delivery (*n* = 14) *n* (%)	*p* *	*p* (FDR)
Breastfeeding (yes/no)	9 (69.2)/4 (30.8)	11 (78.6)/3 (21.4)	0.58	0.725
Sucking problems during breastfeeding (yes/no)	9 (69.2)/4 (30.8)	6 (42.8)/8 (57.2)	0.16	0.266
Artificial ventilation (yes/no)	5 (38.5)/8 (61.5)	2 (14.3)/12 (85.7)	0.15	0.266
Intraventricular hemorrhages (yes/no)	3 (23.1)/10 (76.9)	3 (21.4)/11 (78.6)	0.91	0.91
Unrelenting anxiety during care activities (yes/no)	4 (30.8)/9 (69.2)	8 (57.1)/6 (42.9)	0.15	0.266

* statistical significance at *p*-value < 0.05; FDR, false discovery rate.

**Table 2:** TSM disorders by means of neonatal period complications.

Variable	Over-Responsivity *n* (%)	Under-Responsivity *n* (%)	*p* *	*p* (FDR)
Breastfeeding (yes/no)	16 (80)/4 (20)	4 (57.1)/3 (42.9)	0.244	0.59
Sucking problems during breastfeeding (yes/no)	10 (50)/10 (50)	5 (71.4)/2 (28.6)	0.335	0.59
Artificial ventilation (yes/no)	5 (25)/15 (75)	2 (28.6)/5 (71.4)	0.856	0.59
Intraventricular hemorrhages (yes/no)	3 (15)/17 (85)	3 (42.9)/4 (57.1)	0.134	0.59
Pharmacotherapy (yes/no)	9 (45)/11 (55)	2 (28.6)/5 (71.4)	0.455	0.59
Type of birth (vaginal/vaginal with oxytocin induction/caesarean section)	7 (35)/2 (10)/11 (55)	2 (28.6)/2 (28.6)/3 (42.9)	0.492	0.59

* statistical significance at *p* value < 0.05.

**Table 3:** An association of TSM and sleep difficulties.

Variable	Normal Sleep *n* = 16 *n* (%)	Insomnia *n* = 11 *n* (%)	p *
Under-responsivity *n* = 7	2 (28.6%)	5 (71.4%)	0.08
Over-responsivity *n* = 20	14 (70%)	6 (30%)	

* statistical significance at *p*-value < 0.05.

## Data Availability

Data are available from the corresponding author upon request.

## Supplementary Materials

The following are available online at https://www.mdpi.com/2076-3425/11/3/362/s1, Table S1: Logistic regression of neonatal period variables.
