# Supplementary material for: Tactile Processing and Quality of Sleep in Autism Spectrum Disorders
Source: Brain Sci. 2021 Mar 12;11(3):362. doi: 10.3390/brainsci11030362 (PMC8001965; doi:10.3390/brainsci11030362)
Supplement: Supplementary file 1 [file brainsci-11-00362-s001.pdf]

Table S1. Logistic regression of neonatal period variables.

Tactile under-responsivity				
Variable	Coefficient	Std. Error	OR	P
Age	0.13935	0.18759	1.1495	0.4576
Breastfeeding="YES"	-1.32173	1.05543	0.2667	0.2105
Sucking_problems="YES"	0.26692	1.18796	1.3059	0.8222
Artificial_ventilation="YES"	-0.73821	1.52446	0.4780	0.6282
IVH="YES"	1.79867	1.48059	6.0416	0.2244
